# Paradoxical Air Microembolism Induces Cerebral Bioelectrical Abnormalities and Occasionally Headache in Patent Foramen Ovale Patients With Migraine

**DOI:** 10.1161/JAHA.112.001735

**Published:** 2012-12-19

**Authors:** Eser Başak Sevgi, Sefik Evren Erdener, Mehmet Demirci, Mehmet Akif Topcuoglu, Turgay Dalkara

**Affiliations:** Department of Neurology, Faculty of Medicine, Hacettepe University, Ankara, Turkey

**Keywords:** cerebral ischemia, embolism, migraine, PFO, shunts

## Abstract

**Background:**

Although controversial, paradoxical embolism via patent foramen ovale (PFO) may account for some of the migraine attacks in a subset of migraine with aura (MA) patients. Induction of MA attacks with air bubble injection during transcranial Doppler ultrasound in MA patients with PFO supports this view. It is likely that cerebral embolism in patients with right-to-left shunt induces bioelectrical abnormalities to initiate MA under some conditions.

**Methods and Results:**

We investigated changes in cerebral bioelectrical activity after intravenous microbubble injection in 10 MA patients with large PFO and right-to-left cardiac shunt. Eight PFO patients without migraine but with large right-to-left shunt and 12 MA patients without PFO served as controls. Four MA patients with PFO were reexamined with sham injections of saline without microbubbles. Bioelectrical activity was evaluated using spectral electroencephalography and, passage of microbubbles through cerebral arteries was monitored with transcranial Doppler ultrasound. Microbubble embolism caused significant electroencephalographic power increase in MA+PFO patients but not in control groups including the sham-injected MA+PFO patients. Headache developed in 2 MA with PFO patients after microbubble injection.

**Conclusions:**

These findings demonstrate that air microembolism through large PFOs may cause cerebral bioelectrical disturbances and, occasionally, headache in MA patients, which may reflect an increased reactivity of their brain to transient subclinical hypoxia–ischemia, and suggest that paradoxical embolism is not a common cause of migraine but may induce headache in the presence of a large PFO and facilitating conditions.

## Introduction

An association between patent foramen ovale (PFO) and migraine with aura (MA), as well the indications for PFO closure in migraine, is still debated.^1–4^ Solid epidemiological evidence is lacking to support an association between the 2 conditions. On the other hand, PFO closure studies in migraine patients report conflicting results.^5–8^ Taken together, the available data suggest that PFO is not a common migraine risk factor; however, the possibility that the paradoxical cerebral embolism might trigger migraine attacks in a subset of patients under certain conditions, which may have not been possible to disclose with the epidemiological studies, cannot be excluded at present. Indeed, there are several lines of evidence to suggest that cerebral microembolism can potentially trigger migraine aura and headache. It has been reported by several groups that the intravenous microbubble injection during transcranial Doppler (TCD) ultrasound examination provoked aura and headache in some migraine patients with aura and PFO, accounting for up to 15% of the patients examined in some series.^9–11^ Similarly, cerebral embolism during PFO closure led to migraine attacks shortly after the intervention.^2^ Moreover, a prospective study showed that PFO closure could decrease the frequency and severity of attacks in migraine patients without previous stroke when patients were selected based on magnetic resonance (MR) evidence of cerebral embolism.^6,12^ Improvement in migraine after pulmonary fistula closure in patients with hereditary hemorrhagic telangiectasia further supports the link between cerebral embolism and migraine attacks.^13^ Paradoxical microembolism may therefore be a triggering factor for some attacks in a subgroup of MA patients.^12^ Large PFOs associated with atrial aneurysms or the presence of prothrombotic hematologic factors or conditions that are increasing intrathoracic pressure may predispose these patients to cerebral microembolism, similar to stroke patients with postulated paradoxical embolism.^14–16^ Interestingly, a majority of the cerebellar infarct–like lesions detected on MR images of young migraine patients were located in the vascular border zone territory,^17^ suggesting a microembolic origin,^18^ although the pathology of these MR lesions remains to be verified.

It has also been shown that intracarotid air bubble injection can trigger cortical spreading depressions (CSDs), possibly by causing small brain foci of hypoxia–ischemia in mice.^19^ Similarly, paradoxical microembolism in patients with PFO, when a critical threshold is reached, may trigger a CSD or a CSD-like event and, hence, initiate an aura, which may be followed by headache. Unfortunately, unlike in rodents, CSDs cannot be easily detected with scalp recordings in humans because of a thick cranium and convoluted brain. However, spectral EEG analysis, which can detect subtle alterations in cerebral bioelectrical activity, may disclose the changes induced by microembolism.

In the present study, we evaluated the changes in cerebral bioelectrical activity induced by intravenous bubble injection into patients having high-grade right-to-left shunt as well as MA. We combined EEG recording with concomitant TCD examination to associate changes in bioelectrical activity with air embolism.

## Methods and Patients

### Patients

Twenty-seven healthy MA patients were selected from our neurology outpatient clinic who meet the International Headache Society criteria for typical migraine with aura^20^ and agreed to give consent. Patients with migraine without aura or small and medium-size PFOs were not included in the study. The family history, accompanying diseases and drugs used were recorded (Table 1). The first 7 MA patients had already been diagnosed as having a large PFO with significant right-to-left shunt and had been referred to the study. Subsequently, of the 20 MA patients screened, 6 were diagnosed as having a large PFO, whereas 13 did not have a PFO; hence, they were allocated to the MA with or without PFO groups, respectively. One MA patient who had a small PFO was not included in the study. Because only 1 male patient in each MA group was recruited, they were not included in the final analysis, which limited the study to homogeneous groups of female patients; however, adding data for the male patients to the analysis did not change the statistical significance values. Two MA patients with PFO were not included in the analysis because of excessive EEG artifacts before or after the bubble test. Accordingly, the results are reported for 10 MA patients having a large PFO with right-to-left shunt and 12 MA patients without PFO (first control group). Eight migraine-free control patients, who were diagnosed having a large PFO with right-to-left cardiac shunt by TCD ultrasound or transesophageal echocardiography in our neurology or cardiology department (see Table 1 for details), were selected because they agreed to participate in the study (second control group). Only 1 of these control patients had infrequent tension-type headaches. Four of the MA patients with right-to-left cardiac shunt agreed to participate in a second session, in which they were reexamined while performing the Valsalva maneuver without air bubble injection. Clinical data for all 30 patients in 3 groups included in the study are presented in Table 1, along with their family history, accompanying diseases, and drugs used.

**Table 1.** Clinical Features of Subjects

	Sex	Age, y	Attack Frequency	Headache Severity	Nausea/Vomiting	Photophobia/Sonophobia	Family History	Prophylactic Treatment
MA patients with right-to-left shunt								

Patient 1	F	36	2/wk	M/S	+	+	2 Sisters	–

Patient 2*	F	43	2/wk	M/S	+	+	Mother	Amitriptyline

Patient 3*	F	42	2/wk	M/S	+	+	Mother	–

Patient 4	F	41	3/mo	M/S	+	+	Mother	–

Patient 5	F	30	2/wk	M/S	+	+	Mother and 2 sisters	Valproic acid

Patient 6	F	46	2/wk	M/S	+	+	–	–

Patient 7	F	55	2/wk	M/S	+	+	–	–

Patient 8*	F	17	1/wk	M/S	–	+	–	–

Patient 9	F	26	1/mo	M/S	+	+	Mother	–

Patient 10*	F	24	1/wk	M/S	+	–	–	Flunarizine

MA patients without right-to-left shunt								

Patient 11	F	40	1/2 mo	M/S	+	+	Mother	–

Patient 12	F	26	1/mo	M/S	+	+	Mother	–

Patient 13	F	32	2/wk	M/S	+	+	Mother	–

Patient 14	F	43	2/wk	M/S	+	+	Mother	Valproic acid

Patient 15	F	27	1/mo	Mild	+	+	–	–

Patient 16	F	33	2/wk	M/S	+	+	Mother and 1 sister	–

Patient 17	F	54	2/wk	M/S	+	+	Father and 1 sister	–

Patient 18	F	29	2/wk	M/S	–	+	–	–

Patient 19	F	35	1/mo	Mild	+	+	–	–

Patient 20	F	33	2/mo	M/S	+	+	–	–

Patient 21	F	24	1/3 mo	M/S	+	+	–	–

Patient 22	F	43	3/mo	M/S	+	+	–	–

	Sex	Age	Headache	Chief Complaint/Indication for Neuroimaging or TTE	MR Imaging Findings	Indication for TCD or TEE
Patients without MA but with right-to-left shunt						

Patient 23	M	17	No	Palpitations, shortness of breath	—	TTE suspicious for ASD

Patient 24	M	34	No	Mild cognitive symptoms, forgetfulness	Chronic infarcts in left occipitotemporal area and right centrum semiovale	Multiple embolic infarcts

Patient 25	F	34	No	Transient left-sided hemiparesia and dysarthria	Chronic infarcts in right frontal and temporal cortical and subcortical areas	TIA and multiple embolic infarcts

Patient 26	F	47	No	Transient right-sided paresthesia and hemihypoesthesia	Multiple T2 hyperintense lesions in frontoparietal subcortical areas	TIA and multiple MR imaging lesions

Patient 27	M	33	No	Transient right-sided hemiparesia	Chronic infarcts in left cerebellar hemisphere, cerebellar peduncles, and cerebral hemisphere. Mild cerebral atrophy	TIA and multiple embolic infarcts

Patient 28	M	21	No	Transient left-sided hemiparesia	—	TIA

Patient 29	F	46	No	Transient hemiparesia, bilaterally	Multiple T2 hyperintense lesions in cerebral white matter	TIA and multiple MR imaging lesions

Patient 30	F	33	TTH	Transient left central facial palsy	–	TIA

MA indicates migraine with aura; Mild headache, patient is aware of the headache but is able to continue daily routine; moderate-to-severe headache, headache inhibits daily activities or is incapacitating; M/S, moderate to severe; TTE, transthoracic echocardiography; TCD, transcranial Doppler; TEE, transesophageal echocardiography; ASD, atrial septal defect; TIA, transient ischemic attack; TTH, tension-type headache.

* Patients were examined twice by infusing saline with or without air bubbles while they performed the Valsalva maneuver.

All patients were evaluated for headache by 2 experienced physicians from the neurology department (E.B.S. and S.E.E.), and the patients’ neurological examination was normal. Informed consent was obtained from all patients before the study. The study protocol was approved by the Ethical Committee of the Faculty of Medicine, Hacettepe University.

### TCD and EEG Applications

EEG recordings were performed while the subjects were at rest in the supine position on a comfortable examination chair in a quiet room. A 32-channel EEG amplifier (ISO 1032CE, Braintronics, Almere, the Netherlands) with a custom-developed recording system and software was used for recordings and analyses. A standard electrode cap with 19 electrodes in accordance with the 10–20 system was used. Electrode impedances were kept at <10 KΩ. Forehead reference, 1.6- to 70-Hz band-pass, and 50-Hz notch filters were applied. The sampling rate was 256 Hz.

Multi-Drop X4 (DWL, Sipplingen, Germany) TCD equipment and Spencer Marc 600 (Spencer Technologies, Washington, DC) head probe fixation set and TCD-8 software were used for the microbubble test. The size of the right-to-left shunt was graded, according to the maximum number of microbubbles observed, as a small shunt (3 to 20 microbubbles), large shunt (≥20 microbubbles), the “shower” pattern (uncountable number of microbubbles), and the “curtain” pattern. The curtain pattern refers to a shower of microbubbles that disturb the envelope of spectral configuration. For EEG recording, the TCD transducer was secured on the scalp over the EEG cap. Posterior temporal bone windows were used to insonate the P2 segments of the posterior cerebral artery. Briefly, the P2 segment was insonated at a depth of 60 to 70 mm and confirmed for its close proximity to Rosenthal basal vein, whose flow direction is the same with P2 (away from the probe).

After monitoring of P2 flow, EEG recording was started while the patient's eyes were shut. The Doppler audio signal was then turned off. Following a 10-minute EEG recording, agitated saline (9 mL of saline and 1 mL of air) was injected via an antecubital intravenous catheter while the patient carried out a 5-second–duration Valsalva maneuver. To investigate the effect of air bubbles separate from that of the Valsalva maneuver, a second EEG was recorded in 4 of the migraine patients with PFO, while the patient performed the Valsalva maneuver and received saline infusion but without air bubbles. The time of microbubble transit through P2 was marked on EEG recordings, which were continued for 30 minutes. The spectral EEG analysis was performed in all 34 recordings in 4 groups.

### Power Spectral Analyses

Each subject's continuous multichannel EEG data were segmented into 4-second epochs. Each epoch was visually reviewed, and epochs with artifacts were excluded from the analysis. Linear trends were removed from and a Hanning window was applied to every epoch of each channel. The power spectra of all epochs were computed using the fast Fourier transform technique and were ensemble averaged separately for the epochs preceding and those following microbubble injection. Preinjection power spectra were used as baseline. The decibel change in power (ΔP) induced by microbubble injection was calculated for each electrode location with the following formula: ΔP=10×log (postinjection power/baseline power). ΔP was calculated for the total spectral range (4 to 45 Hz) and for each of the theta (4 Hz<theta≤8 Hz), alpha (8 Hz<alpha≤13 Hz), beta (13 Hz<beta≤30 Hz), and lower gamma (30 Hz<lower gamma≤45 Hz) bands. Only the power in the lower gamma band (30 to 45 Hz) rather than the entire band (30 to 60 Hz) was evaluated because a 50-Hz hardware notch filter had been used during recordings. Frequencies <4 Hz were not included in the analyses because of their susceptibility to low-frequency noises such as eye-blink and movement artifacts. Additionally, the global total power (sum of the total power at all electrode locations) was calculated for each subject.

### Statistical Analysis

Global total ΔP was compared between patient groups by Kruskal–Wallis ANOVA followed by multiple comparisons of mean ranks (STATISTICA 8.0, Statsoft Inc, Tulsa, Okla). Global total ΔP with and without microbubble injections in the subgroup of MA patients with PFO, who were reexamined for evaluation of the effect of Valsalva maneuver, was compared by *t* test for dependent variables.

For age, 3 groups were compared with Kruskal–Wallis variance analysis. The headache frequency was compared between MA groups with and those without PFO by Mann–Whitney *U* test, and headache severity, presence of family history, and use of prophylactic medication use were compared with use of the Fisher exact test. Mean values are given with their standard errors in the text.

The 1-way design in this study with 3 groups having sample sizes of 12, 10, and 8 achieves a power of 91.6%, using the Kruskal–Wallis test with a target significance level of 0.05 to significantly detect a difference of 0.6 dB between groups. This power calculation based on 2000 samples obtained by Monte Carlo simulation assumes normal distribution of ΔP values with a 0.4-dB SD, forcing a nonparametric approach due to small and unequal sample sizes.

## Results

The mean (±SE) ages of MA patients with PFO, MA patients without PFO, and PFO patients without migraine were 36±4 (n=10), 35±3 (n=12), and 33±7 (n=8) years, respectively, and there were no statistically significant differences (*P*=0.90). Six MA patients with PFO and 6 MA patients without PFO had a migraine history in their first-degree relatives (*P*=0.691). The headache frequency was 6±0.9 per month for MA patients with PFO and 4±1 for MA patients without PFO (*P*=0.189). The pain intensity was graded subjectively as mild or moderate to severe, depending on whether the patient's typical headache inhibited daily activities. Ten MA patients with PFO and 10 MA patients without PFO had moderate to severe headache (*P*=0.480). Three patients in the MA with PFO group but only 1 patient in the MA without PFO group were receiving prophylactic medication; however, this unequal distribution was not significantly different (*P*=0.293). Some clinical features of MA patients are given in Table 1.

On infusion of agitated saline, an intense microbubble embolism through the P2 segment of the posterior cerebral artery was observed in either the shower (8 of 10 subjects in the MA with PFO group and 7 of 8 subjects in the PFO without MA group) or the curtain (2 subjects in the MA with PFO group and 1 subject in the PFO without MA group) pattern. Shortly after microbubble injection, headache developed in 1 MA patient with PFO. This 41-year-old woman had 3 migraine attacks per month on average. She was not using any prophylactic treatment and she had a family history of migraine. After microbubble injection, the curtain pattern was detected in her TCD record. Shortly after visualization of microbubbles in the posterior cerebral artery, the patient complained of restlessness and felt as if she was going to have a migraine attack. Approximately 5.5 minutes later, the patient's headache started and continued throughout the EEG recording. Her headache was lateralized to the right side, and she described it as similar to but milder than her usual migraine headaches. She had no nausea or vomiting, photophobia, or sonophobia. She did not need pain medication and returned to her busy professional practice, so the exact duration of headache could not be determined. A second patient, a 24-year-old woman with no family history of migraine, complained of headache without aura after the bubble test. She usually had 1 migraine attack per week and was receiving flunarizine prophylaxis. Approximately 10 minutes after visualization of microbubbles with the curtain pattern, she started to have a throbbing headache on both sides and became anxious. She had moderate nausea. She did not report photophobia or sonophobia. The intensity of headache increased toward the end of EEG recording, and she needed to take oral acetaminophen for relief. She described the headache episode as similar to her typical migraine attacks. None of the other patients reported similar discomfort after bubble injection.

### Electrophysiological Findings

**Figure 1.**
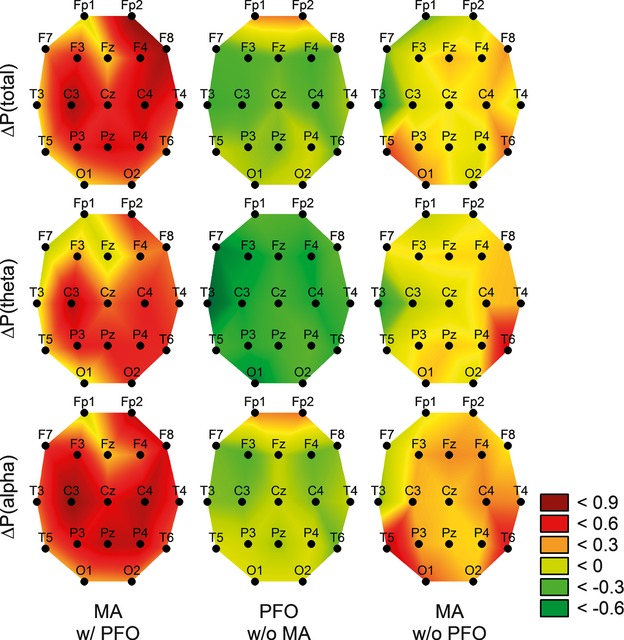
Cerebral air embolism induced EEG power changes only in MA patients with PFO, not in PFO patients without migraine or MA patients without PFO. Emboli-induced spectral power changes (ΔP) were calculated for each electrode location by subtracting the baseline spectrum from the postinjection spectrum in each subject and were averaged for every group (columns). ΔP values (in decibels) are color-coded, with higher values depicting an increase. Changes in the power for total spectra (top rows) and for theta (middle rows) and alpha (bottom rows) bands are illustrated separately. MA indicates migraine with aura; PFO, patent foramen ovale.

**Figure 2.**
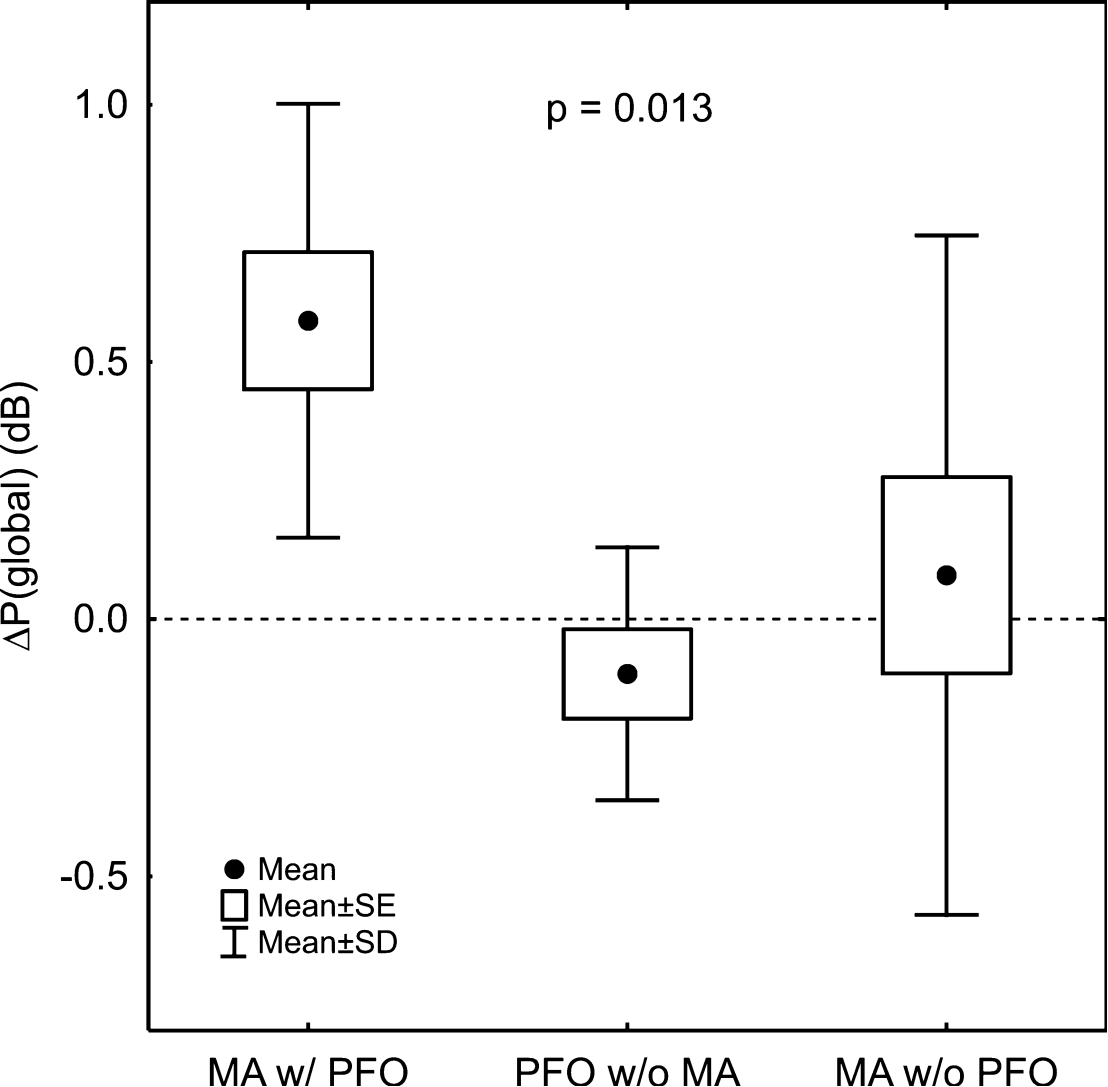
Mean global changes in total EEG spectral power induced by microbubble injections for each group. Total spectral power differences at all electrode locations were summed to obtain the global changes (decibels) for each patient and then the group averages were calculated. MA w/ PFO group was significantly different from the other groups (P=0.013). MA indicates migraine with aura; PFO, patent foramen ovale.

We found that after the bubble injection, the EEG power increased in all spectral bands and electrode positions in the MA with PFO group (global total power change was 0.580±0.133 dB), whereas it remained essentially unchanged in patients with right-to-left shunt but without migraine (−0.107±0.087 dB) despite the presence of equally intense embolic showers observed in their cerebral arteries (Figures 1 and 2). No significant differences between preinjection and postinjection spectra were observed in MA patients who did not have PFO (0.085±0.190 dB), strongly suggesting that the EEG changes were induced by cerebral air embolism but not by the Valsalva maneuver or by the anxiety of the procedure. The differences among the 3 groups were statistically evaluated by comparing the global total power change calculated by summing the power changes at all electrode locations for each subject (Figure 2). Group means were significantly different for power changes (*P*=0.013). The power increase in the MA with PFO group was significantly different than that for the other groups (*P*=0.045 compared with the MA without PFO group, *P*=0.009 compared with the PFO without migraine group), whereas the difference between the PFO without MA and MA without PFO groups was not significant (*P*=0.612).

**Figure 3.**
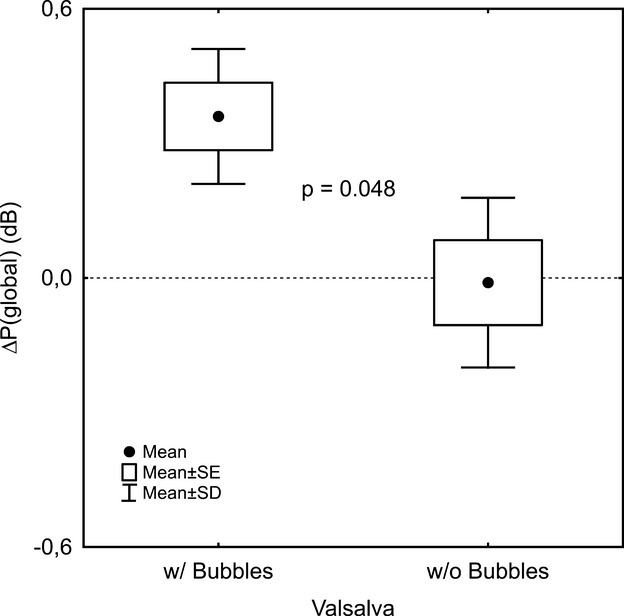
EEG changes induced in MA patients with PFO were not due to the Valsalva maneuver but rather to air embolism. To differentiate a possible effect of Valsalva maneuver–induced intracranial pressure changes from the effect of microbubbles on EEG power, 4 of the MA patients with PFO were reexamined by infusing saline without air bubbles while patients were performing the Valsalva maneuver. Mean values of the global total EEG spectral power changes after injections with and without bubbles are shown for these 4 patients. Injections with bubbles resulted in significantly higher power changes (*P*=0.048). MA indicates migraine with aura; PFO, patent foramen ovale.

For the 4 MA patients with PFO who were reevaluated by infusing saline without bubbles while they performed the Valsalva maneuver, the global total ΔP was significantly higher when the ΔP values recorded after the bubble test in the first session were compared with those for the bubble-free saline infusion (0.360±0.075 vs −0.010±0.095 dB, *P*=0.048) (Figure 3).

The power increase in the MA with PFO group was prominent in alpha and theta bands. ΔP showed similar increases in beta and gamma bands; however, these changes were inconsistent across the subjects in all groups. These 2 upper frequency bands have low amplitude, constituting only a small percentage of the total power, and thus are more prone to noise than are the lower frequency bands. The low signal/noise ratio might have caused the inconsistency.

These changes were independent of any alterations in vigilance, which was carefully controlled by delivering verbal stimuli to the patient every 3 to 4 minutes and by observing the clinical and EEG manifestations. Moreover, because the baseline and postinjection EEG recordings were obtained in the same single session, some of the factors that might confound the results, such as anxiety during the recordings or an uncomfortable sleep the previous night, were minimized.

## Discussion

We found that only MA patients with large PFOs developed significant spectral power changes after intravenous microbubble injection. No significant EEG abnormalities were induced in control patients with right-to-left cardiac shunt but not migraine, although their TCD examination clearly demonstrated the transit of microbubble showers through cerebral arteries. Similarly, MA patients who did not have PFO displayed neither cerebral bioelectrical changes nor air embolism after microbubble injection. The EEG changes observed in MA patients were not related to the Valsalva maneuver or to alterations in vigilance, which was closely monitored with verbal stimuli during recording and by carefully scanning the vigilance-associated EEG changes during analysis. Because PFO patients with and without MA had equally large right-to-left shunts, allowing intense cerebral microembolism after the intravenous bubble injection, the spectral EEG changes observed in MA patients may reflect an increased sensitivity of their brain to transient subclinical hypoxia–ischemia induced by air emboli. No EEG changes were observed in MA patients without PFO or in MA patients with PFO who were retested without agitated saline while performing the Valsalva maneuver, suggesting that the EEG changes detected in MA patients with PFO were indeed induced by air embolism to the susceptible MA brain but not by Valsalva maneuver. All MA patients had a history of visual auras, suggesting that their occipital lobes were prone to develop CSD or CSD-like bioelectrical abnormalities. Genetic factors may underlie such susceptibility, as suggested by prevalent family history in MA patients. Importantly, clinical phenotype characterized with headache appeared in only 2 patients, suggesting a continuum of microembolism-induced changes ranging from clinically and electrophysiologically silent brief hypoxic–ischemic episodes to transient bioelectrical abnormalities corresponding to an intermediary stage and, rarely, headache at the more severe end of the spectrum.^21^

We do not know the cellular correlates of EEG disturbances induced by air emboli, although our data clearly indicate that the brain of MA patients is sensitive to embolism-induced perturbations. Supporting this idea, studies in mice report that the bioelectrical disturbances are correlated with the intensity of microembolism-induced hypoperfusion.^19^ The EEG literature is rich with reports describing the changes recorded in stroke patients, but these come from cases with lasting brain lesions. Unlike long-lasting metabolic perturbations,^18,22^ the impact of very brief embolism on cerebral bioelectrical activity has not been studied even in experimental animals except for the recent study reporting that air emboli induce CSD in the mouse.^19^ Unfortunately, however, CSDs are very difficult to detect in humans through surface recordings. The thick cranium and propagation of the spreading depolarizing wave in various directions due to cortical convolutions, in addition to common DC artifacts during EEG recordings, preclude a reliable detection of the characteristic DC potential shift or spreading EEG depressions with surface recording. Therefore, we may have failed to detect the CSD in the patient who had aura after bubble injection. In the majority of MA patients who did not report an aura after microbubble injection, the metabolic perturbation must have been severe enough only to alter the EEG spectrum without reaching the threshold to trigger CSD.

We observed unambiguous differences between the cerebral bioelectrical responses of MA patients with large PFO and nonmigraineurs to air embolism. Although it is not clear how a parenchymal event in the human brain can trigger a migraine attack, genetic susceptibility and environmental factors may cause the extracellular K^+^ and glutamate levels to sufficiently rise and initiate CSD or a CSD-like depolarizing event.^23^ In fact, several studies have suggested that interictal cortical excitability may be increased in migraineurs.^24,25^ However, this genetic susceptibility is translated into a clinical phenotype only in the presence of certain facilitating conditions such as estrogen withdrawal or sleep deprivation. Hence, the brain of MA patients appears to have a propensity to develop bioelectrical disturbances that can be elicited by factors known as migraine triggers. These abnormal bioelectrical activities may be followed by headache if they are sufficiently intense to activate and sensitize trigeminal nerve endings on the neighboring pial vessels.^26^ Cerebral microembolism appears to be one of the rare triggers in migraine patients with large right-to-left shunts, although alternative mechanisms such as access to the brain of vasoactive substances bypassing the filtering in lungs have also been proposed.^12^ A support for embolism has come from a recently published prospective randomized, sham-controlled clinical trial.^6^ This study enrolled patients with large right-to-left shunts, moderate to severe migraine, and no previous symptomatic cerebrovascular events but with brain MR imaging lesions presumably due to silent ischemic episodes. Reported results included a significant reduction in the frequency and severity of migraine attacks by PFO closure. Not surprisingly, migraine attacks in PFO patients can be triggered by other factors as well; the latter possibility may account for the negative results of some PFO closure studies in which patients were not selected with criteria suggesting paradoxical cerebral embolism. Importantly, however, the negative studies and the persistence of migraine attacks to varying degrees even in patients who benefit from the PFO closure clearly indicate that PFO closure cannot be used for the treatment of migraine attacks without establishing the causal relationship for individual patients.

In conclusion, this study demonstrates that the intravenous injection of air bubbles induces bioelectrical disturbances in the brain of MA patients who have large right-to-left cardiac shunts, unlike in patients with equally large right-to left cardiac shunts but without migraine. However, the majority of these patients did not experience headache, as commonly observed on TCD with air-contrast studies,^9–11^ suggesting that subclinical hypoxia–ischemia induced by paradoxical cerebral embolism is not a common cause of migraine attacks in MA patients with large PFOs but may occasionally trigger aura and headache in the susceptible MA brain under the presence of physiologically facilitating conditions and/or when the clearance of the embolus is delayed.
